# Drug repurposing: a nexus of innovation, science, and potential

**DOI:** 10.1038/s41598-023-44264-7

**Published:** 2023-10-19

**Authors:** Maria Cristina De Rosa, Rituraj Purohit, Alfonso T. García-Sosa

**Affiliations:** 1 Institute of Chemical Sciences and Technologies “Giulio Natta” (SCITEC) – CNR, L.go F. Vito 1, 00168 Rome, Italy; 2 Structural Bioinformatics Lab, CSIR-Institute of Himalayan Bioresource Technology (CSIR-IHBT), Palampur, HP 176061 India; 3 Academy of Scientific and Innovative Research (AcSIR), Ghaziabad, 201002 India; 4 Department of Molecular Technology, Institute of Chemistry, University of Tartu, Ravila 14a, 50411 Tartu, Estonia

**Keywords:** Drug discovery and development, Biotechnology

## Abstract

The urgency of finding therapeutic solutions for emerging and existing health challenges has never been more pronounced. In the pursuit of this goal, the value of a strategy that makes use of existing resources is being recognized: drug repurposing or repositioning of compounds for new indications. Such approaches are employed against cancer, rheumatoid arthritis, multiple sclerosis, HIV/AIDS, and many other diseases. This Collection, aptly titled “Drug Repurposing”, includes research and perspectives from scientists at the forefront of this innovative field.

## Reinvigorating the drug discovery pipeline

In the traditional drug discovery pipeline, the journey from an idea to an approved therapy is long, costly, and fraught with uncertainty. The failure rate is high, and the financial and temporal investments are significant. Drug repurposing is an alternative strategy that allows to reduce the time and costs of pharmaceutical research in which new uses are identified for drugs already approved or under investigation. In the last decades, many successful examples of repositioning have been reported for the treatment of different pathologies^1^. Through the strategic redirection of existing drugs, molecules, or compounds that have already passed safety tests, in fact, drug repurposing or repositioning accelerates the pace of discovery. The research presented in this Collection illuminates various facets of experimental and computational drug repurposing, reflecting its complexity, potential, and burgeoning maturity.

## A Tapestry of techniques and targets

What strikes the reader of this Collection is the diversity of techniques and targets that may be involved in drug repurposing. From machine learning (ML)-driven frameworks for kinase inhibitor repositioning, to the utilization of natural products as potential therapeutics against viral infections, the methodologies are as varied as they are inventive. The Collection also showcases the integration of multi-disciplinary sciences. Computational techniques, pharmacological insights, and molecular biology interweave to form a cohesive picture of a promising and dynamic field.

## Leveraging artificial intelligence

Several papers in this Collection highlight the role of artificial intelligence (AI) and machine learning in drug repurposing. These advanced computational methods allow researchers to sift through vast amounts of data, identify hidden patterns, and generate insights that would be very difficult to uncover through traditional means. The KUALA framework is a prime example, automating the identification of kinase active ligands and prioritizing multi-target scores for best repurposable molecules^2^. Emphasizing the high similarity of kinase binding sites, the research underscores its dual role in drug selectivity and poly-pharmacology. Leveraging this similarity, De Simone et al. explore the potential for drug repositioning on analogous targets^2^. KUALA, which employs 12 different machine learning methods for classification, successfully assigned kinase inhibitors currently in clinical trials, to each of the known targets in 84% of cases.

In two other papers published as part of this Collection, Hetmann et al.^3^ and Pirolli et al.^4^ successfully combined physics-based computational methods with deep learning models showing how science adapts and evolves to meet the challenges of drug repurposing. The novel computational pipeline CavitomiX^3^, based on active site cavity comparisons, was capable of identifying inhibitors for selected target enzymes. This technology offers a novel drug repurposing approach, independent of structure and sequence alignments, identifying two approved drugs, fusidic and flufenamic acid, as exhibiting anti-viral activity against SARS-CoV-2. Notably, the established computational pipeline can be quickly modified to address new pathogens.

## Expanding the scope of repurposing

Scientists contributing to the Collection address diverse therapeutic needs. This Collection illustrates how repurposed drugs can be explored for COVID-19, Alzheimer’s disease (AD), and infectious diseases. Parolo et al. present a comprehensive study on AD, for which, despite significant investments in drug development, only one disease-modifying treatment has been recently approved^5^. The researchers introduce a single cell-led systems biology pipeline for identifying drug repurposing candidates. By leveraging single-cell RNA sequencing data from brain tissues of AD patients, genome-wide association study results, and multiple gene annotation resources, they constructed a multi-cellular AD disease molecular network. This network provided cell-specific insights into AD pathophysiology and identified 54 candidate drugs, primarily targeting MAPK and IGF1R signaling pathways, for potential AD therapy.

Another study addressed the urgency to discover solutions for antibiotic resistance with innovative approaches that explore the therapeutic potential of natural products and their synthetic derivatives^6^. These efforts reflect a broader shift in thinking, where drug repurposing is not merely a tactic but a holistic strategy to respond to global health concerns.

Schake et al. delve into the role of the gut microbiota in modulating the effects of dietary polyphenols on human health^7^. The study highlights the bidirectional interactions between polyphenols and gut microbiota, emphasizing the importance of microbial metabolism in determining the bioavailability and bioactivity of polyphenols. The researchers provide insights into the potential health benefits of polyphenols, including anti-inflammatory, antioxidant, and anti-cancer properties. They also discuss the challenges in studying polyphenol-microbiota interactions and suggest future research directions to harness the therapeutic potential of dietary polyphenols.

## Navigating challenges

The path to successful drug repurposing is not without challenges. Issues of selectivity, toxicity, and the intricate balance between binding site similarity and the number of targets are just a few of the complex considerations that must be navigated. The provision of experimental results can, as ever, help to make predictions stronger and better focused and explainable. Despite this, a drug may be effective in a different application case, but at doses that render it unusable or ineffective for another indication due to issues of efficacy or side-effects, the most common obstacles to drug approvement^8^. However, there is a long and solid history of off-label use of pharmaceutical products in the clinic for indications other than the primary or listed case. Another avenue for possible development is finding new targets that have not been described or targeted yet but may have clinical use such as in the illuminating the druggable genome project^9^ with compounds that are already known to be safe. In addition, companies are in some cases donating some of their libraries or collaborating with institutions such as the DNDi to help make compounds accessible for vector-based diseases^10^. The research within this Collection does not shy away from these opportunities and challenges, providing valuable insights and analytical rigor to guide future efforts.

## References

[1] Pushpakom S, Iorio F, Eyers P (2019). Drug repurposing: progress, challenges and recommendations. Nat. Rev. Drug Discov..

[2] De Simone G, Sardina DS, Gulotta MR (2022). KUALA: A machine learning-driven framework for kinase inhibitors repositioning. Sci. Rep..

[3] Hetmann M, Langner C, Durmaz V (2023). Identification and validation of fusidic acid and flufenamic acid as inhibitors of SARS-CoV-2 replication using DrugSolver CavitomiX. Sci. Rep..

[4] Pirolli D, Righino B, Camponeschi C (2023). Virtual screening and molecular dynamics simulations provide insight into repurposing drugs against SARS-CoV-2 variants Spike protein/ACE2 interface. Sci. Rep..

[5] Parolo S, Mariotti F, Bora P (2023). Single-cell-led drug repurposing for Alzheimer’s disease. Sci. Rep..

[6] Rossiter S, Fletcher M, Wuest W (2017). Natural products as platforms to overcome antibiotic resistance. Chem. Rev..

[7] Schake P, Dishnica K, Kaiser F (2023). An interaction-based drug discovery screen explains known SARS-CoV-2 inhibitors and predicts new compound scaffolds. Sci. Rep..

[8] Yang H, Sun L, Li W (2018). In silico prediction of chemical toxicity for drug design using machine learning methods and structural alerts. Front. Chem..

[9] Nguyen DT, Mathias S, Bologa C (2017). Pharos: Collating protein information to shed light on the druggable genome. Nucleic Acids Res..

[10] Drugs for Neglected Diseases initiative, https://dndi.org/research-development/treatments-delivered/. Last website visit 22.09.2023.

